# What can multimodal data tell us about online synchronous training: Learning outcomes and engagement of in-service teachers

**DOI:** 10.3389/fpsyg.2022.1092848

**Published:** 2023-01-06

**Authors:** Jun Xiao, Zhujun Jiang, Lamei Wang, Tianzhen Yu

**Affiliations:** ^1^Shanghai Engineering Research Center of Open Distance Education, Shanghai Open University, Shanghai, China; ^2^Department of Educational Technology, School of Education, Shanghai Normal University, Shanghai, China

**Keywords:** engagement, eye-tracking, facial expression, EEG, in-service teacher training

## Abstract

Teachers’ engagement in online learning is a key factor in improving the effectiveness of online teacher training. This paper introduces a multimodal learning analytics approach that uses data on brain waves, eye movements and facial expressions to predict in-service teachers’ engagement and learning outcomes in online synchronous training. This study analyzed to what extent the unimodal and multimodal data obtained from the in-service teachers (*n* = 53) predict their learning outcomes and engagement. The results show that models using facial expressions and eye movements data had the best predictive performance on learning outcomes. The performance varied on teachers’ engagement: the multimodal model (integrating eye movements, facial expressions, and brain wave data) was best at predicting cognitive engagement and emotional engagement, while the one (integrating eye movements and facial expressions data) performed best at predicting behavioral engagement. At last, we applied the models to the four stages of online synchronous training and discussed changes in the level of teacher engagement. The work helps understand the value of multimodal data for predicting teachers’ online learning process and promoting online teacher professional development.

## Introduction

The COVID-19 pandemic has strongly boosted the development of online learning, which, however, does not prove very effective because the students are poorly motivated and engaged due to untimely feedback, lax supervision, and other factors (Nasir and Ngah, 2022). At the same time, teachers are facing great challenges in their professional development as the transition from traditional education to online learning will cause in them mental changes in relation to their identity as educators and their ideas about education (Wang et al., 2010; Teräs, 2014; Richardson and Alsup, 2015). As teachers must get prepared for online teaching in a short period and quickly grasp the methods and skills needed, providing effective training on professional development for them is of great importance. Advances in educational technology and online learning platforms and changes in educational modes (from offline to online) have made online teacher professional development (OTPD) possible and popular (Parsons et al., 2019; Nami, 2021). OTPD is defined as a format of teacher professional development (TPD) that provides teachers with continuous learning through ICT media (e.g., asynchronous, synchronous, blended or other forms of courses, seminars or learning modules provided online), without having to meet in person with their trainers/instructors and peers each time (Rogers, 2001; Ansyari et al., 2022). It offers a more flexible and personalized way of learning for teachers that overcomes geographical barriers (Chen et al., 2009; Ross, 2011; Powell and Bodur, 2019). Besides, it was found no differences between in-person TPD and OTPD in terms of teacher perceptions and learning outcomes (Ansyari et al., 2022).

Previous studies on OTPD usually focus on online learning efficiency from the perspective of technology application and management (Wang et al., 2010). But as a matter of fact, technology *per se* cannot promote TPD – that can only be realized by further clarifying the relation between technology and TPD. Engagement is an effective predictive indicator of long-term learning performance (Camacho et al., 2020). There is a growing body of research that demonstrates the importance of engagement for learning and achievement. In the field of teacher professional development, engagement is a key dimension in ensuring that teachers receive a complete training program, and some studies have shown that high levels of engagement in training help teachers apply the knowledge and skills they have learned to their practice after the training is complete (Holmes et al., 2021). According to Fredricks et al. (2004), the integration of behavior, emotion, and cognition under the concept of engagement is valuable because it can provide a richer characterization of learning than single-component studies. With the growing importance of online teacher training in recent years, researchers have turned to focus on teachers’ engagement in the online learning environment. For instance, Liu and Zhang (2021) found that teachers generally think online learning is not interactive enough, giving it an average score of only 2.36 out of 5 on the interactive level, i.e., interaction with peers and instructors. Philipsen et al. (2019) found that timely support and feedback can help teachers specify their learning needs and improve training efficiency. In other words, compared with asynchronous learning, synchronous and blended learning methods can better stimulate the teachers’ enthusiasm and engagement in the online training program.

There are three ways to measure engagement – self-report questionnaire, data mining based on learning logs, and sensor-based technology (Appleton et al., 2006; Sinatra et al., 2015; Camacho et al., 2020). In the past, the research on learning engagement is generally focused on the measurement of single dimensions. This includes evaluating only behavioral engagement based on postures such as hand raising, note-taking, and head propping (Liao et al., 2019; Vanneste et al., 2021), or evaluating emotional and cognitive engagement in a VR environment (Dubovi, 2022) while ignoring behavioral engagement. But in fact, engagement should not be evaluated by separate dimensions (Sharma and Giannakos, 2020). In addition, as Cleary and Zimmerman (2012) pointed out, engagement is essentially a continuous process that fluctuates in time as students become immersed in learning, so it’s necessary to measure the learners’ engagement in a dynamic way. In this respect, some researchers noted that the grain size of engagement measures can range from the micro level (e.g., individual engagement in the present moment, task, or learning activity) to the macro level (e.g., a group of learners in a class, course, school, or community) and suggested that at the micro level, engagement can be measured using physiological and psychological indicators such as brain imaging, eye tracking, response time, or attention allocation (Broughton et al., 2010; Sinatra et al., 2015; Dubovi, 2022). In the field of teacher professional development, we found that more and more research is focusing on teachers’ learning processes, especially incorporating physiological and psychological data. For example, Chang et al. (2018) explored teachers’ emotional experiences by coding the nonverbal expressions of their recorded videos. Wolff et al. (2016) investigated differences in how expert and novice teachers perceive problematic classroom scenes with eye-tracking technology. In addition, some researchers have also focused on using data such as facial expressions to evaluate the quality of teachers’ teaching for their professional development (Zheng et al., 2020). It can be seen that currently in the field of TPD, while objective data channels are receiving increasing attention from researchers, multimodal data are less explored.

These days more researchers have come to analyze multimodal data because they, compared with unimodal data, can integrate subjective (e.g., self-report questionnaire) and objective data, and enable the capturing of the cognitive, emotional and behavioral learning process (Sinatra et al., 2015) from multiple perspectives. Cognitive engagement reflects the use of deep learning strategies, involving the integration of new information and existing knowledge. In measuring cognitive engagement, electroencephalogram (EEG), a neuroimaging technology, can capture the total activities of all nerve cells simultaneously oscillating in the learning process (Niedermeyer and da Silva, 2005). Studies have shown that the four patterns of EEG frequency are strongly related to emotional and cognitive states (Hassib et al., 2017). Baceviciute et al. (2020) used EEG to capture the learners’ cognitive process in VR learning and found that VR learners displayed a higher level of Theta activities in the parietal lobe, which implied the possible use of long-term memory coding, searching, and other cognitive approaches. The visual attention data channel is another objective means of identifying fluctuations in cognitive engagement during learning. Bixler and D’Mello (2016) used eye-tracking sensors to record general eye gaze indicators, such as the number of fixations, fixation durations, variability in fixation durations, and saccade lengths, to measure wandering during computerized reading. Moreover, galvanic skin response (GSR), heart rate (HR) are also used to measure cognitive load (Cranford et al., 2014; Larmuseau et al., 2020) and concentration (Cooper et al., 2006; Sharma et al., 2020).

Regarding emotional engagement, it refers to the learner’s emotion-related states during learning activities, such as happiness, enjoyment, boredom and frustration (D’Mello et al., 2017). Facial expressions are mostly used to measure and predict emotional engagement, for instance, when learners interacted with a game-based learning environment, Taub et al. (2019) captured seven facial expressions (i.e., joy, sadness, disgust, contempt, surprise, fear, anger) of learners and combined them with a traditional self-report questionnaire to portray the dynamics of learners’ emotional engagement. Behavioral engagement refers to a person’s behaviors of efforts and contributions during a learning activity (Fredricks et al., 2004). Since the mind–body connection suggests that observable physical responses can be used to infer unobservable mental states (D'Mello et al., 2017), some researchers have collected data such as human-computer interactions based on gamified learning environments to measure behavioral engagement (Psaltis et al., 2018). In a synchronous learning environment, non-verbal cues such as facial expressions, gestures and body postures captured from video image frames of classroom data can be used to effectively identify unobtrusive behavioral engagement (Whitehill et al., 2014; Ashwin and Guddeti, 2019).

According to Ning and Downing (2011) and Liu and Zhang (2021), learning experience can be interpreted as the learner’s interaction with the teaching and learning environment, leading to the acquisition of subject-related knowledge or the development of personal/professional skills. The previous studies of learning prediction focused on identifying risk learners by using online learning data to predict dropout rate (Costa et al., 2017; Moreno-Marcos et al., 2020) and paid little attention to the learning experience. Nowadays, more researchers and scholars are paying attention to learners’ interests, motivation, engagement, and other indicators, the development of which is greatly beneficial for improving self-directed learning in the learners and improving the teaching process (Wang et al., 2022). It is equally important because it helps improve the learning experience. Furthermore, it is possible to precisely predict a series of learning indicators with sensor-captured data, including data on emotions, eye movements, brain waves, GSR, or various combinations of them (Emerson et al., 2020; Olsen et al., 2020; Sharma et al., 2020). However, these studies are mostly focused on human-computer interaction learning environments, such as gamification environments (Giannakos et al., 2019; Emerson et al., 2020) and human-robot interaction (Cui et al., 2022) with little attention to computer-assisted collaborative learning scenarios. Olsen et al. (2020) divided the students into groups of two and used multimodal data to predict collaborative learning outcomes. This is an innovative study that broadens the scope of the application of Multimodal Learning Analytics (MMLA) in collaborative learning. As a matter of fact, a key element of OTPD is collaborative and interactive learning among teachers, which also holds the key to adult learning (Powell and Bodur, 2019).

Generating data on teachers’ behavioral patterns, cognitive processes, as well as emotional experiences, has the potential to help develop and refine more effective pedagogy and support tools for use in informal and formal teacher professional development opportunities. At present, although researchers have explored many data stream combinations, few studies in the field of OTPD have ever examined the relation between unimodal and multimodal data to understand their synergetic effects and ability to explain the teachers’ performance in the test and other critical indicators (e.g., engagement). But that is what’s vitally important because as adult learners, the teachers’ online learning outcomes are also affected by multiple factors. For instance, “time sequence” plays an important role in interactions and communication during online synchronous learning, and it has been used to analyze interactions among fellow learners (Chen et al., 2009). This is to say that when online course designers guide the trainee teachers to study by themselves, discuss or make reports, the sequence of doing all that will affect the learners’ degree of concentration and other aspects. Therefore, predictive analytics can help the designers understand the teachers’ engagement and other experiences in online learning, which is of great importance for promoting self-regulated learning (SRL; Sharma et al., 2020). Although multimodal data has shown great potential in the field of education, its ability to serve as a means of understanding and improving teachers’ learning processes remain largely unexplored. To better leverage the design capabilities of multimodal data, we need to evaluate the effectiveness of multimodal data. This paper systematically assesses how different data streams can benefit predictive analytics. Our findings quantify the expected benefits of using various multimodal data from physiological sensing and help advance research in the area of learning technologies.

### Research objectives and research questions

In this paper, we build predictive models on the learners’ eye gaze, facial expressions of emotions, brain waves, self-report engagement, and test of knowledge points in an effort to make up for the scarcity of literature in OTPD.

We aim to (a) build predictive models of different modal combinations and examine the precision of unimodal and multimodal models, including data acquisition, data preprocessing and model training. Specifically, data acquisition refers to the acquisition of learner brainwave timing data, eye movement timing data, and facial timing data. The data acquisition and pre-processing module are used to acquire the temporal data of brainwave, emotion, and eye movement over time as well as the questionnaire data. The preprocessing part completes the process of data cleaning, data purification, and time calibration to obtain the unified standard online learning temporal data under multimodality. The training model refers to the multimodal analysis system, which takes raw brainwave, eye movement and expression data as multimodal input data and questionnaire data as indicators to extract the features of multimodal input data and trains them to generate prediction models of different indicators. Among them, the feature engineering module adopts the form of automatic machine mining to realize dynamic feature extraction, feature filtering, feature correlation analysis with questionnaire big data, and feature principal component analysis for online learning temporal data.

(b) use the models to predict in-service teachers’ changing engagement in the learning process. The feature engineering segments the temporal data according to the teaching design, and further feature extraction is performed for each segment of data. The data modeling and analytical inference module models the time-series data according to its features with participation and knowledge tests, which can be used to infer the indicators within each period. The stage prediction refers to using the indicator model to make predictions for the input data in different periods to get each indicator within different periods. This includes metrics for groups and metrics for individuals.

For these goals, we have three research questions.

1. Does multimodal data provide more precise predictions than those gained by unimodal data for engagement?
2. How well do combinations of brain waves, facial expressions and eye gaze predict the engagement of in-service teachers?
3. What are the features of learner engagement according to the prediction model?

## Materials and methods

### Participants, experimental design, and procedure

The participants in this study included 56 in-service teachers who were enrolled in a teacher training program in Shanghai, China. Participants had not previously attended a training program related to ClassIn. During the experiment, data about three teachers were invalid because of falling headbands or other reasons, which gave us valid data on 53 teachers for further analysis. There were 28 males and 25 females; 80.4% of them were aged 20–40 and 19.6% were 40–50. The participant’s personal information will be kept confidential, and only their ID, testing score, and the captured data will be maintained. They will be notified of the data collection and asked to sign the Informed Consent Form (ICF).

The training course – Online teaching based on ClassIn – is selected for this experiment. ClassIn is a useful online class system that has been used in the schools of many localities across China. It has a rich pool of functions, but many teachers do not know how to use it to facilitate or improve their instruction, which is why the researchers decide to provide training on this subject. This course is focused on how to do online teaching through ClassIn and contains four main aspects:

(1) Critical view of online teaching, (2) Instruction on online teaching and ClassIn, (3) Experience with ClassIn, (4) Feedback and Reflection.

**Figure 1:**
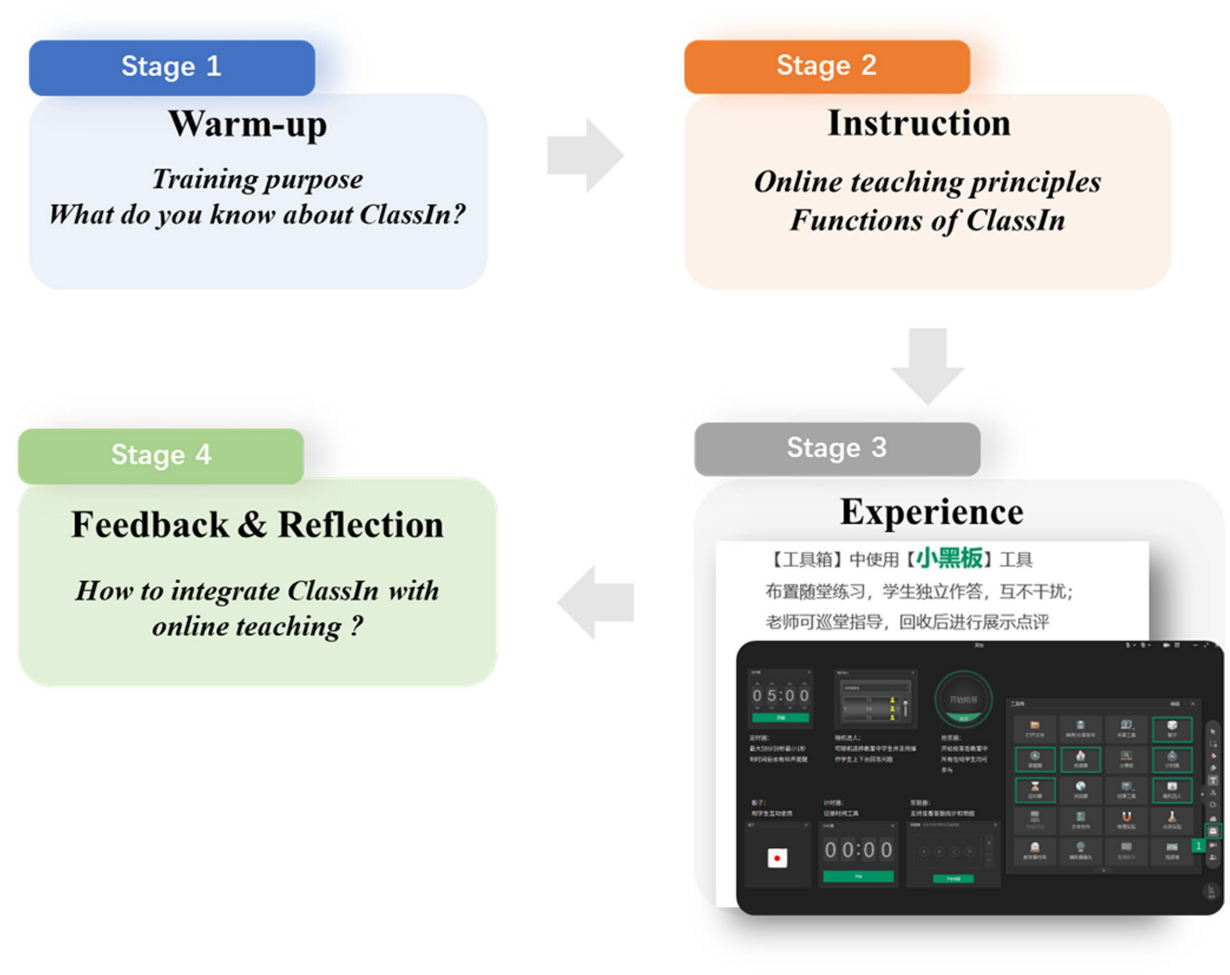
The procedure of the training program.

In light of the features of adult learning (Ke and Xie, 2009; Abedini et al., 2021), the course centers on collaborative tasks and involves four stages (Figure 1). The first stage is an introduction, in which the instructor, by sharing real cases, introduces what will be taught and urges the teachers to think and share their views on online teaching during the pandemic. In the second stage, the instructor will introduce the functions of ClassIn, such as the *Group Discussion Function*. The third stage features collaborative learning, in which teachers are divided into several groups to practice with ClassIn, e.g., preparation before class, interaction and feedback during class. The fourth stage is for feedback and reflection, in which the instructor takes the teachers to review and reflect on what they have learned.

The research design of our study is a single-group time series design that involves repeated measurement of a group (Ross and Morrison, 2004). The experimental protocol consists of three sessions and took a total of 75 min. 10 min before the experiment started help participants calibrate an eye tracker, EEG device, and facial expressions of emotions software. This study used BrainCo headbands called Focus 1 (Focus, BrainCo, 2022), a wearable EEG device with 3 hydrogel electrodes, to collect and analyze the EEG data at 160 Hz *via* Wi-Fi (Kosmyna and Maes, 2019). As to facial expressions, we first turned on cameras to record the facial expressions, then Facereader software was used to analyze the data (Terzis et al., 2013). Eye tracker (Tobii T120) was used to capture gaze data. Once calibration was completed, participants began the training course session which took 40 min. After the course, participants filled in a questionnaire and knowledge test that took 25 min.

### Measures

The questionnaire, adapted from the ones developed by Deng et al. (2020) and Liu et al. (2010), concerns engagement – cognitive, emotional and behavioral (Fredricks et al., 2004). A Cronbach’s alpha of 0.801 indicates good reliability of the questionnaire. Of the three indicators, cognitive engagement includes seven items (e.g., “I think about the relation among different knowledge during online learning”) (0.833), emotional engagement includes six items (e.g., “I enjoy the atmosphere of online synchronous training” and “I like online synchronous training”), and behavioral engagement includes seven items (e.g., “When I have a question, I’d ask the instructor and fellow teachers through the chat box of the live streaming platform”). The questionnaire uses a five-point Likert scale, from “strongly agree” to “strongly disagree,” a higher score indicates a higher level of reported engagement.

The knowledge test is designed by the research team (research members have long years of experience in teacher training) according to the contents of the course to understand how well the teachers have grasped what they are taught. There are single-choice questions (six items, 36 points), and multi-choice questions (eight items, 64 points) with a total score of 100. The test measured retention and also the comprehension and transfer of knowledge. For example, one of the questions asked the basic functions of ClassIn “What are the forms of assignments that can be submitted online in ClassIn.” Another example is about the deep understanding of online teaching “What principles to keep in mind when students study in groups online.” Analysis of the knowledge results indicated a good internal consistency score, with a Cronbach’s alpha of 0.76.

### Multimodal data collection and pre-processing

During the study, we captured participants’ knowledge test scores. In addition, we collected sensor data from three different sources: EEG, facial expression, and eye-tracking.

#### EEG

To study brain wave values, we based our research on prior research that reported using consumer EEG headbands with 1–6 channels (Andujar and Gilbert, 2013). This study used the Focus band (Focus 1, BrainCo, 2022), co-developed by scientists from Harvard’s center of brain science, to collect EEG features from the participants, which has also been used to detect engagement in previous studies (Kosmyna and Maes, 2019). According to the International 10–20 electrode placement system, one electrode is located at the FPz position, as well as the reference and ground electrodes of TP9. Neural oscillations *α* (7–11 Hz), *β* (11–20 Hz), and *θ* (4–7 Hz) were collected and normalized to EEG values between 0 and 100, and a higher value indicates higher attention. We use one data extracted every 10 s for analysis. After pre-processing, the EEG data of each learner was output with Time and EEG values.

#### Facial expression

To study the participants’ facial expressions, we used FaceReader, a video-based facial expression tracking system (Noldus, 2019), to analyze the facial expressions of the teachers or learners. The analytical system is a reliable, professional software used for automatic facial expression analysis that can tell seven basic emotions: neutral, delight, surprise, sorrow, anger, fear, and disgust. Its working principles are as follows:

(a) Face finding: an algorithm based on deep learning is used to find human faces;
(b) Face modeling: nearly 500 key points are used to produce precise artificial face models;
(c) Face classification: an artificial neural network is used to classify the expressions.

Seven expressions are identified every time, each scored with a floating-point number ranging from −1 to 1. First, we removed empty or failed results. Second, we screened off those not obtained during the experiment (according to the official start and finish time). Then we selected the highest of the seven numbers as the facial expression of that very moment and classified it with a number from one to seven. The results of facial expression identification came out every 0.2 s, and we sampled them every second considering the huge amounts of data. We also noticed that the software did not export anything when the expression remained unchanged, and only exported a record with a corresponding time when it changed. Therefore, after we sampled the expressions by second, we filled up the lost values to make sure there was a facial expression score for every second. The expression data of all teachers (*n* = 53) put together constituted 3,901 pieces of time-sequenced records. Then we used tsfresh, a Python package for systematic feature engineering from time series and other sequential data, to extract 779 static features, which were imported into the models for training.

#### Eye tracking

To capture the trajectory of eye movements for analysis, we used Tobii T120 eye tracker to record where every participant looked on the screen during the course and how far their eyes were from the screen. Before the training, each participant was required to make adjustments for sitting position and distance. First, the researcher will turn on the eye-tracking test function of the eye-tracking device, and two dots will appear on the screen to indicate the gaze points of the left and right eyes. By micro-adjusting the seat distance to ensure that each participant’s gaze point is at a close uniform level, the gaze range is just the entire learning material. The software recorded the position and distance-to-screen of both the left and right eyes and formed six-dimensioned data (Table 1).

**Table 1:** Set of GAZE features.

Feature	
EyePosLeftX	Horizontal offset from screen, left eye gaze
EyePosLeftY	Vertical offset from screen, left eye gaze
EyePosLeftZ	Distance from screen, left eye
EyePosRightX	Horizontal offset from screen, right eye gaze
EyePosRightY	Vertical offset from screen, right eye gaze
EyePosRightZ	Distance from screen, right eye

We compared the time-sequenced data of eye movement trajectory with videos of the online course to match where the eyes gazed at and the duration of gaze with each frame. Then we designed polygonal interested areas that involved about four to six interested targets, and determined whether the captured eye movement trajectory (only horizontal and vertical position was considered) was in that area as a high-level description of the teachers’ eye gaze. The proportion of how long they gazed at each interested area was recorded as an important indicator of interest or engagement.

At the same time, the six-dimensioned data were imported into tsfresh as representing the eye movement trajectory to extract features, and altogether 779 static features were obtained, which, when applied to follow-up model training, led to six major features. The six major features were then combined with features describing the gaze at the interested areas, which gave us a collection of features reflecting the eye movement trajectory, and that was used for the next step of multimodal model training.

### Multimodal predictive modeling

**Figure 2:**
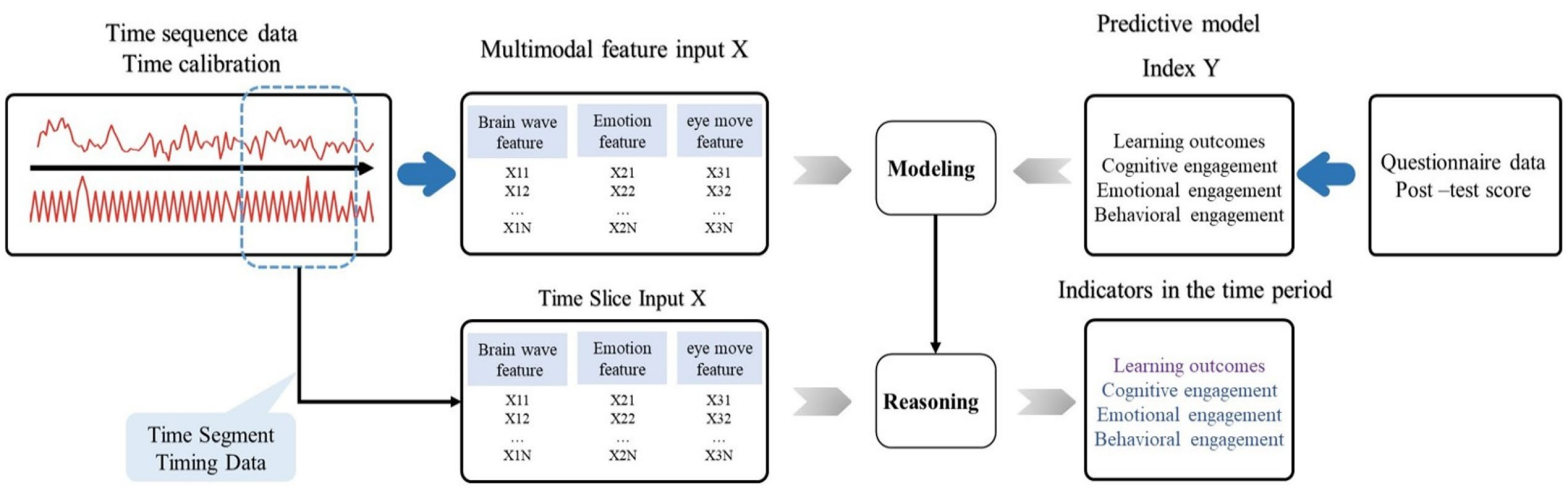
Multimodal data streams and predictive modeling approach.

To study how well the multimodal data can predict the teachers’ learning feedback, we developed a multimodal learning prediction model based on brain waves, facial expressions, and eye-gaze trajectory, and designed the questionnaire surrounding four analytical targets: cognitive engagement, emotional engagement, behavioral engagement, and learning outcomes. For constructing the model, this study uses an analysis method based on time series data features (Emerson et al., 2020; Olsen et al., 2020), by extracting features of time series data, filtering features, and analyzing the main features to obtain multiple sets of features. These feature values can be used as the input sample independent variables X of the model, i.e., brain wave feature, emotion feature, eye move feature. The dependent variables are derived from the questionnaire. Specifically, reported cognitive engagement, affective engagement, behavioral engagement, and test-based learning outcomes were used as the target Y for the study analysis, and a model from X to Y was constructed and trained to predict the target value Y for each stage of analysis (Figure 2). Facial expressions were labeled in seven different categories to create a time-sequenced series, from which we extracted 779 general features. The movement trajectory of the left and right eye (X, Y, Z) went through feature engineering to generate 779 general features too, so did the time-sequenced brain wave data. The three sets of features – totaling 2,337 – were imported into the decision tree as independent variables, while the scores of every target in the questionnaire were the dependent variables. To prevent over-fitting, they were imported into the training model, with 70% as the training set and 30% as the test set (Kang and Oh, 2020). The allocation ratio was set after several tests based on the best training fit. Each analytical target corresponded with a model, so four targets, and four multimodal predictive models. At that time, all data on the entire course were divided according to the four stages, and the four models were used to, respectively, predict each of the four targets in each stage.

During the experiment, we made the hypothesis that there must be some major features among the general ones that were strongly relevant to brain waves, facial expressions, and eye movement trajectory. So we extracted major features related to the predictive targets during model training, and imported them, as descriptive of brain waves, facial expressions, and eye movement trajectory, into the models for training again, which produced multimodal predictive models based on major features. Different multimodal experiments generated different multimodal major features (about 4–10 of them), which meant the latter was data-sensitive.

## Results

We use tsfresh to extract the features of temporal data, and import the obtained features to Decision Tree Classifier for training, Decision Tree Classifier comes from sklearn (an open source python language-based machine learning library), then sklearn’s classification report function was used to automatically calculate precision, recall, F1. Precision is a measure of result relevancy while recall is a measure of how many truly relevant results are returned. The F1 score is reported as an agglomerative measure between precision and recall. The focus of this study is on the precision of the prediction model, so we choose it as the key indicator (Sharma et al., 2020).

To answer research question 1 and 2, we investigated how well unimodal and multimodal models (e.g., EGG, facial expression, and eye gaze) could predict the teachers’ engagement. As shown in Table 2, the predictive model integrating data on eye movements, facial expressions and brain waves is the most precise (0.65) in predicting cognitive engagement, with the highest Recall (0.67) and F1 score (0.64) as well. That is higher than the scores of unimodal prediction and bimodal prediction.

**Table 2:** Cognitive engagement prediction results.

Data used	Precision	Recall	F1
Gaze	0.36	0.43	0.40
EEG	0.26	0.48	0.33
Face	0.40	0.57	0.46
Gaze + EEG	0.43	0.48	0.45
EEG + Face	0.44	0.39	0.39
Gaze + Face	0.47	0.43	0.45
Gaze + Face + EEG	**0.65**	**0.67**	**0.64**

Bold values represent best performance.

The researchers also found that the multimodal predictive model integrating data on eye movements, facial expressions and brain waves had the highest precision (0.61), Recall (0.67) and F1 score (0.52) in predicting emotional engagement too, but that does not mean more modal data would naturally lead to higher predictive precision. For instance, the unimodal model using only brain wave data has a precision of 0.47, higher than any bimodal data combinations (see Table 3).

**Table 3:** Emotional engagement prediction results.

Data used	Precision	Recall	F1
Gaze	0.27	0.35	0.47
EEG	0.47	0.48	0.48
Face	0.24	0.23	0.28
Gaze + EEG	0.25	0.33	0.24
EEG + Face	0.35	0.30	0.43
Gaze + Face	0.31	0.30	0.42
Gaze + Face + EEG	**0.61**	**0.67**	**0.52**

Bold values represent best performance.

Table 4 shows that multimodal predictive models are more precise than unimodal models in predicting behavioral engagement, with the model combining data on facial expressions of emotions and eye-gaze being most predictive with a precision of 0.75. Of unimodal models, the one using facial expressions data is most predictive with a precision of 0.43, while that using EEG data performs worst with a precision of only 0.17.

**Table 4:** Behavioral engagement prediction results.

Data used	Precision	Recall	F1
Gaze	0.43	0.38	0.39
EEG	0.17	0.38	0.24
Face	0.49	0.52	0.60
Gaze + EEG	0.28	0.24	0.25
EGG + Face	0.56	0.62	0.59
Gaze + Face	**0.75**	**0.52**	**0.60**
Gaze + Face + EGG	0.70	0.62	0.62

Bold values represent best performance.

Table 5 shows that as far as learning outcomes are concerned, the predictive model combining data on eye movements and facial expressions has the highest precision of 0.66 – higher than the model integrating data on eye movements, facial expressions, and brain waves. We also found that of unimodal models, the one using eye movements data has the highest precision of 0.52 whereas that using brain wave data has the lowest precision of 0.29.

**Table 5:** Learning outcomes prediction results.

Data used	Precision	Recall	F1
Gaze	0.52	0.48	0.44
EEG	0.29	0.24	0.26
Face	0.35	0.33	0.32
Gaze + EEG	0.56	0.43	0.41
EGG + Face	0.45	0.36	0.39
Gaze + Face	**0.66**	**0.43**	**0.41**
Gaze + Face + EEG	0.52	0.55	0.53

Bold values represent best performance.

**Figure 3:**
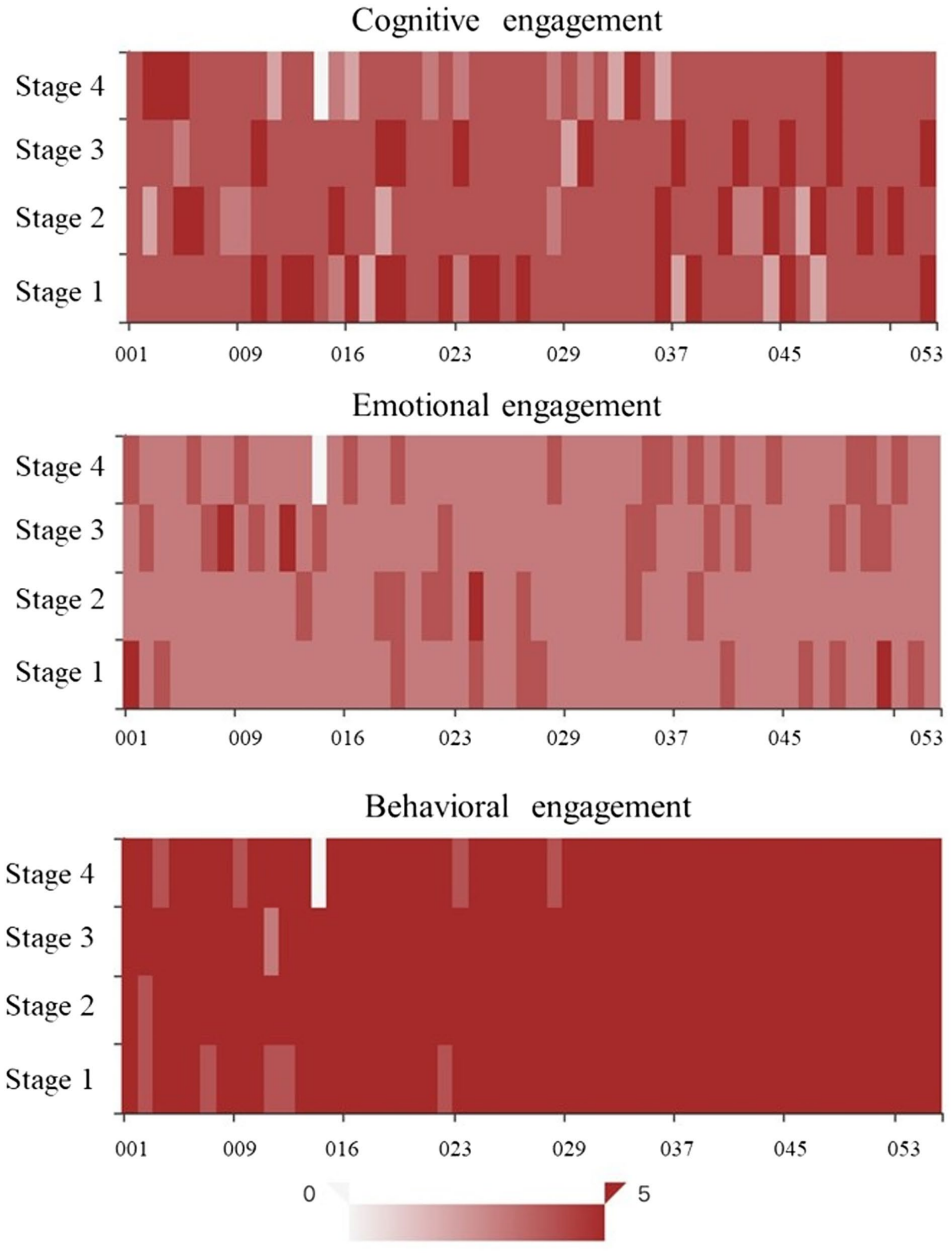
Thermal distribution map of multi-modal fusion characteristic data.

To answer question 3, we exported the scores of the learners’ emotional, cognitive and behavioral engagement in the four learning stages. As shown in Figure 3, a thermal distribution map of multi-modal fusion characteristic data was used to evaluate the engagement of each stage, a darker color means a predicted score higher and closer to 5, and a lighter color means a score lower and closer to 0. Generally speaking, learners have the highest score on behavioral engagement and the lowest on emotional engagement. As to the change of engagement through four stages, cognitive engagement wanes first and waxes later. In the first stage, for example, the instructor aroused the teachers’ interests by presenting a research report on the current status of online teaching and sharing real cases, and urged them to reflect and contemplate on the common problems occurring in their classes. Behavioral engagement waxes first and wanes later. In the second and third stages, the teachers discussed specific topics and solved problems collaboratively, including research, sharing of views, and group report, which stimulated their learning enthusiasm. Emotional engagement wanes first and waxes later. The highest score in the third stage indicates the highest emotional engagement during collaboration and interaction, which is consistent with the questionnaire results – teachers are generally more interested in collaborative learning.

**Figure 4:**
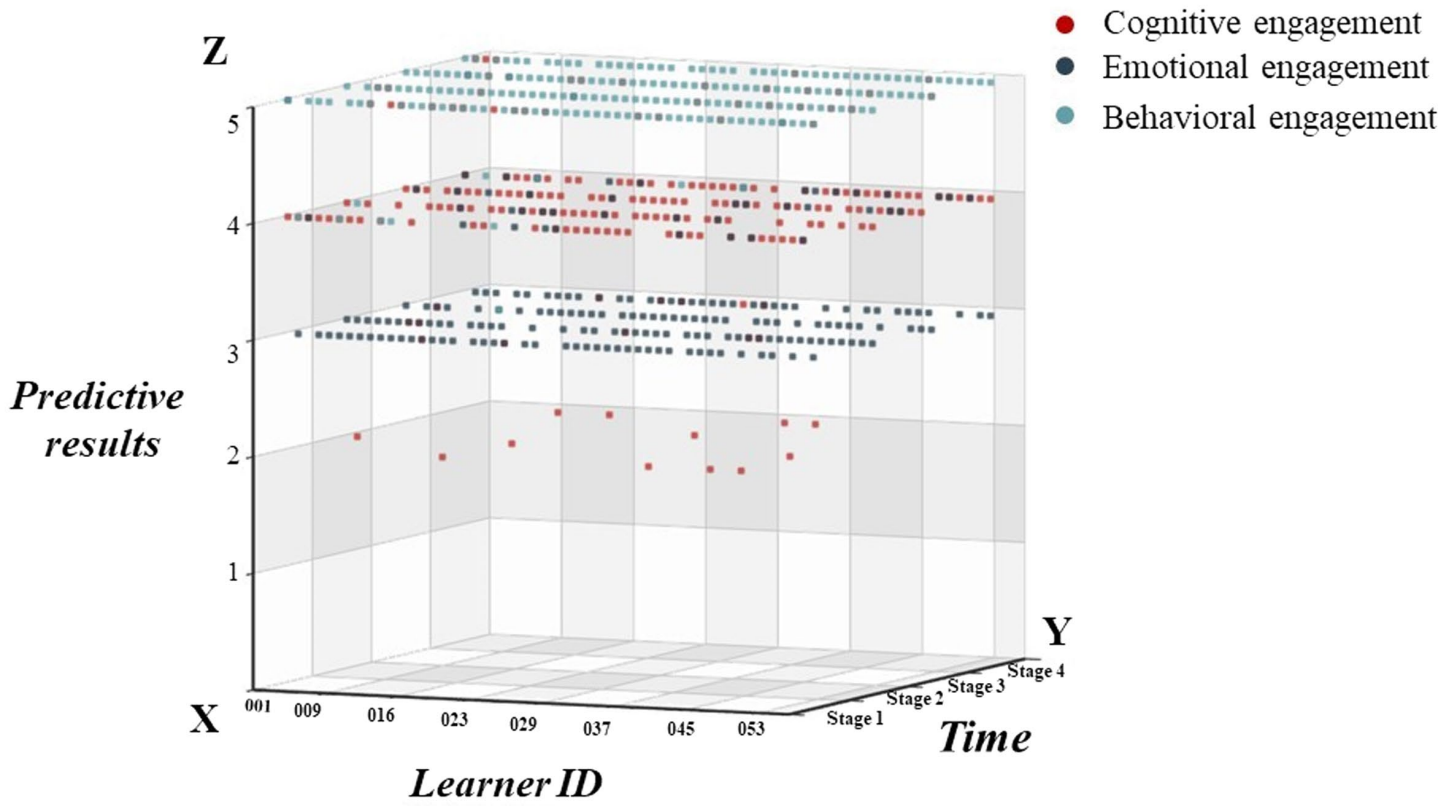
Spatial and temporal distribution coordinates of engagement.

As shown in Figure 4, researchers have developed 3D coordinates for engagement based on the predictive models. The X axis represents the learners’ serial number, Y axis the four learning stages, and Z axis the predictions on cognitive, emotional and behavioral engagement. The coordinates can reflect how each learner’s engagement changes through the four stages. We found that most learners maintain a high level of behavioral engagement through the stages with little change. Predictions on their emotional engagement show that most of them have a low level of emotional engagement at first, but some see it increasing over time. Their cognitive engagement changes rather drastically, and it drops significantly in the third and fourth stage for a few learners.

## Discussion

Improved quality of instruction contributes to better student learning achievement (Ansyari et al., 2022). Teachers, as adult learners, must seek self-improvement constantly to promote professional development and embrace changes. That’s why designing and planning high-quality teaching training for teachers is highly important (Creemers et al., 2012; Carrillo and Flores, 2020). Learning analytics is a key approach to refining the teaching process. Although the teachers’ learning indicators can be explained with different data streams, one important question is how to merge the data obtained from various channels to provide a better, more comprehensive picture of the learning process (Chango et al., 2021). With the rapid development of artificial intelligence such as sensor technology and machine learning, it is possible to capture the participants’ subconscious emotions (Vanneste et al., 2021) and use multimodal data to predict online learning process. In this paper, we extracted the features of multimodal data for training and generated predictive models for different indicators. To be specific, using an analytic method suited to the features of time-sequenced data, we extracted and filtered the features of time-sequenced data on brain waves, facial expressions, and eye movements analyzed the major features, and obtained multiple feature sets, which can be imported into the models as sample X. We took the indicators in the engagement questionnaire and knowledge test as target Y, and developed models matching X with Y for training to predict the target value of Y at each time period.

Addressing RQ1 (Does multimodal data provide more precise predictions from those gained by unimodal data for engagement?), we see that multimodal models are generally more precise than unimodal models on predicting engagement and learning outcomes. However, there are some differences in the predictive results for the three sub-dimensions of engagement. On the one hand, we found that the trimodal prediction model integrating data on facial expressions, eye movements, and brain wave is most precise regarding cognitive engagement and emotional engagement, while the bimodal prediction model that combines facial expression and eye movement data has the best predictive performance in terms of behavioral engagement and learning outcomes. On the other hand, we found that the predictive model integrating Omni-modal data does not always produce the best predictions, which is consistent with the conclusions of previous studies (Emerson et al., 2020). One possible reason is that the excessive noises have undermined the model’s robustness.

That brings us to our second research question, RQ2 (How well do combinations of brain waves, facial expressions and eye gaze predict the engagement of in-service teachers?). First, from the predictive results of cognitive engagement, we found that multimodal predictive models perform better than any unimodal model in prediction, and specifically, we found that the model using EEG data alone is least satisfactory. Cognitive engagement includes psychological positioning, cognitive efforts, and the thinking or attention aroused during the learning activity (Greene et al., 2015). In fact, an imbalance in cognitive understanding, if not properly addressed, may lead to emotional frustration. This may explain why the unimodal model using facial expression data does better than that using eye movement data or using EEG data in predicting cognitive engagement. Second, in terms of emotional engagement, although the most frequently used method to measure emotional engagement without disrupting the learners is analyzing their facial expressions, which helps capture their subconsciously fast-changing emotions (Taub et al., 2019; Vanneste et al., 2021), this study found that the predictive model using only data on facial expressions does not perform well, whereas the trimodal model has the best predictive performance. We also found that the unimodal model using EEG data only is the second most precise in predicting emotional engagement, better than other bimodal or unimodal models, which is inconsistent with a previous study by Soleymani et al. (2016), whose data results demonstrated that facial expression performs better than EEG data. The possible reason may be that our study was based on a real online learning environment. We did not provide learners with videos specifically selected as emotional arousal stimuli to cover the entire emotional range as previous studies have done. Third, this paper found that the model combining data on eye movements and facial expressions is the most precise in predicting behavioral engagement, but adding EEG data into the model would lower its precision. The facial expressions gave better prediction performance, which supports the findings of previous studies (Ashwin and Guddeti, 2019). In sum, the results add to a gap in the field of related research in the past, where many studies have confirmed that facial expression and gaze contribute to identifying, monitoring, and classifying behavioral engagement (D’Mello et al., 2017; Alkabbany et al., 2019), but few studies have focused on how well the predictions work in the combined model.

Addressing RQ3 (What are the features of learner engagement according to the prediction model?), this study showed that the highest score of teachers’ cognitive engagement in the third learning stage. This consists with previous researchers’ conclusions that cognitive engagement is essentially a process of continuous fluctuation that occurs when the person interacts with a specific scenario. When teachers interact with specific learning tasks or environments, cognitive engagement happens (Li and Lajoie, 2021). Helme and Clarke (2001) identified three interacting factors that influence cognitive engagement: the individual, the learning environment, and the task. In the third stage, the online synchronous training environment provides a platform for cognitive engagement where teachers work in small groups to collaborate around specific tasks, which helps stimulate deeper strategies and efforts. However, it is noteworthy that a few learners in this study had significantly decreased cognitive engagement in the third and fourth learning stage. The third stage of training in this study is the collaborative learning stage, where the learners’ emotional and cognitive processes become more complex in an online collaborative learning environment because each group member’s reaction affects the overall emotional climate and learning process (Törmänen et al., 2021; Ye and Zhou, 2022). One possible reason for this is that in this study, we allowed teachers to choose their topics for collaborative inquiry, but lacked scaffolding to facilitate deep reflection and cognitive processing and due to time constraints, some teachers exhibited relatively more low-level cognitive processes (e.g., understand; Lin et al., 2014). In other words, instructors can also appropriately clarify collaboration requirements and evaluation criteria to help learners with self-regulation and self-control (Dabbagh and Kitsantas, 2004).

As to emotional engagement, the predictions also show that of the three dimensions of engagement, the score of teachers’ emotional engagement is the lowest but it increases gradually. This means as the learning activity proceeds, especially after the teachers are divided into groups, they get a stronger sense of belonging and consequently display a higher emotional engagement (Ulmanen et al., 2016). However, the overall score on this dimension remains low may because they are not familiar with each other or may not all be interested in the training theme. Previous research has found a significant relationship between the perceived value of feedback and the emotional engagement with feedback during online learning. Therefore it is not enough to provide feedback during an activity, it is also important to understand how teachers perceive the feedback they receive (Mayordomo et al., 2022).

The results also show that of the three dimensions of engagement, teachers’ behavioral engagement has the highest score as well as the highest prediction in the second and third stages. The Expectancy-Value-Cost Model of Motivation suggests that perceived task value directly influences choice, persistence, and performance, and that engagement translates motivation into action (Barron and Hulleman, 2015), therefore, it is important to support teachers in finding value and relevance in their training (Wigfield et al., 2015). This implies that collaborative learning may be an important way of raising their behavioral engagement in online learning, as teachers may display various interactive behaviors with the contents, materials, and fellow teachers, such as research, communication, and division of work. In addition, we found that higher behavioral engagement does not necessarily represent a higher cognitive process. In other words, higher behavioral engagement may be predominantly low-level cognitive processes (e.g., memorization and comprehension; Ye and Zhou, 2022).

The findings of the study may contribute to the empirical and theoretical development of online teacher professional development. First, many studies have emphasized that online teacher training is beneficial to promote teachers’ professional development. Our study quantifies the predictive and explanatory ability of multimodal data on teachers’ online learning process, which can help advance online learning platforms to design and optimize online courses in the future. Second, this study focuses on an important indicator of teachers’ online learning, namely engagement. In particular, our findings reaffirm that engagement is a fluctuating variable, and we find large differences in teachers’ engagement in training across cognitive, emotional, and behavioral dimensions, as revealed by multimodal data, rather than the traditional use of questionnaires at the post-test. Third, the fact that this study found differences in the variation of teachers’ engagement across instructional activity designs promotes our thinking about how to design sequences of instructional activities to improve the effectiveness of teacher training, especially regarding collaborative learning among teachers.

There are some limitations to this research which also can be considered for future research. First of all, the questionnaire is designed in such a way that the participants, out of habit, would prefer moderate answers to radical ones such as “strongly agree” or “strongly disagree.” As a result, the models have no access to fringe scenarios and are therefore not good at predicting them. Wider samples should be considered in the future to enrich our findings, and the “think aloud” approach can also be adopted to examine and improve the validity of inferring data on the behavioral trajectory. Secondly, to not disturb the teachers during learning, we mainly used the usual data on brain waves, eye movements, and facial expressions for this experiment, but data on more dimensions can be incorporated in the future to expand and enrich the predictive models. Thirdly, as our findings indicate an inclination among the teachers to choose longer-term TPD (Philipsen et al., 2019), follow-up studies can be conducted going forward at greater depth by, for instance, collecting multimodal data on the teachers when they sign up for weeks-long, months-long or even year-long online training. Finally, this study found differences in sub-dimensions of engagement through a predictive perspective, the next step is to conduct a more in-depth analysis of the interplay between cognitive process, emotion, and behavioral engagement in conjunction with the predictive model. Besides, It is critical to help improve teacher training programs based on predicted effects, so that in the future, training course content and processes can be optimized in conjunction with design-based research methods.

## Conclusion

The development of artificial intelligence, including sensor technology, has provided the means to collect and analyze learning data from various channels and to make the predictive models on learners’ engagement and test performance more precise. This information has shed light on how to improve the approach to online teacher training and develop self-adaptive tools. Previous studies have shown the prospects of multimodal data in predicting learners’ learning performance in human-computer interaction, but in the field of OTPD, hardly any researcher has ever noticed the synergizing potential of multimodal data for online synchronous learning.

It is against such a background that this paper created predictive models using various data combinations to examine and evaluate how precise the predictions on learners’ engagement and test performance are. Unlike previous studies that only focused on one or two dimensions of engagement, we developed predictive models for all three dimensions – cognitive engagement, emotional engagement, and behavioral engagement – separately. The results show that by and large, models using bimodal or multimodal data are more precise in predicting engagement, but more modal data does not necessarily result in higher predictive precision. This study tries to make a predictive analysis of the learners’ learning process based on the predictive models, which can reflect the real-time change of their engagement, as we found that the learners’ cognitive engagement, emotional engagement, and behavioral engagement all displayed different features in different learning stages.

## Data availability statement

The raw data supporting the conclusions of this article will be made available by the authors, without undue reservation.

## Ethics statement

Ethical review and approval was not required for the study on human participants in accordance with the local legislation and institutional requirements. The patients/participants provided their written informed consent to participate in this study. Written informed consent was obtained from the individual(s) for the publication of any potentially identifiable images or data included in this article.

## Author contributions

JX and ZJ: conceptualization, writing—review and editing, writing—original draft preparation, and methodology. JX: project administration and funding acquisition. LW: investigation process and data collection. TY: formal analysis. All authors contributed to the article and approved the submitted version.

## Funding

This study was supported both by Shanghai Science and Technology Innovation Action Plan International Cooperation project “Research on international multi language online learning platform and key technologies (no. 20510780100)” and Science and Technology Commission of Shanghai Municipality research project “Shanghai Engineering Research Centre of Open Distance Education (no. 13DZ2252200).”

## Conflict of interest

The authors declare that the research was conducted in the absence of any commercial or financial relationships that could be construed as a potential conflict of interest.

## Publisher’s note

All claims expressed in this article are solely those of the authors and do not necessarily represent those of their affiliated organizations, or those of the publisher, the editors and the reviewers. Any product that may be evaluated in this article, or claim that may be made by its manufacturer, is not guaranteed or endorsed by the publisher.

## References

[ref1] Abedini A. Abedin B. Zowghi D. (2021). Adult learning in online communities of practice: a systematic review. Br. J. Educ. Technol. 52, 1663–1694. doi: 10.1111/bjet.13120

[ref2] Alkabbany I. Ali A. Farag A. Bennett I. Ghanoum M. Farag A. (2019). Measuring student engagement level using facial information. in IEEE International Conference on Image Processing (ICIP), 3337–3341.

[ref3] Andujar M. Gilbert J. E. (2013). “Let's learn! Enhancing user's engagement levels through passive brain-computer interfaces,” in *CHI'13 Extended Abstracts on Human Factors in Computing Systems*. eds. Mackay W. E. Brewster S. A. Bødker S. (New York, NY, USA: Association for Computing Machinery), 703–708.

[ref4] Ansyari M. F. Groot W. De Witte K. (2022). Teachers’ preferences for online professional development: evidence from a discrete choice experiment. Teach. Teach. Educ. 119:103870. doi: 10.1016/j.tate.2022.103870

[ref5] Appleton J. J. Christenson S. L. Kim D. Reschly A. L. (2006). Measuring cognitive and psychological engagement: validation of the student engagement instrument. J. Sch. Psychol. 44, 427–445. doi: 10.1016/j.jsp.2006.04.002

[ref6] Ashwin T. S. Guddeti R. M. R. (2019). Unobtrusive behavioral analysis of students in classroom environment using non-verbal cues. IEEE Access 7, 150693–150709. doi: 10.1109/ACCESS.2019.2947519

[ref7] Baceviciute S. Mottelson A. Terkildsen T. Makransky G. (2020). “Investigating representation of text and audio in educational VR using learning outcomes and EEG,” in Proceedings of the 2020 CHI Conference on Human Factors in Computing Systems (CHI’20). New York, NY, USA: Association for Computing Machinery, 1–13.

[ref8] Barron K. E. Hulleman C. S. (2015). “Expectancy-value-cost model of motivation,” in *International Encyclopedia of the Social & Behavioral Sciences*. ed. Wright J. D. (Oxford), 503–509.

[ref9] Bixler R. D’Mello S. (2016). Automatic gaze-based user-independent detection of mind wandering during computerized reading. User Model. User-Adap. Inter. 26, 33–68. doi: 10.1007/s11257-015-9167-1

[ref10] BrainCo (2022). Available at: https://www.brainco.tech (Accessed December 1, 2022).

[ref11] Broughton S. H. Sinatra G. M. Reynolds R. E. (2010). The nature of the refutation text effect: an investigation of attention allocation. J. Educ. Res. 103, 407–423. doi: 10.1080/00220670903383101

[ref12] Camacho V. L. Guía E. D. L. Olivares T. Flores M. J. Orozco-Barbosa L. (2020). Data capture and multimodal learning analytics focused on engagement with a new wearable IoT approach. IEEE Trans. Learn. Technol. 13, 704–717. doi: 10.1109/TLT.2020.2999787

[ref13] Carrillo C. Flores M. A. (2020). COVID-19 and teacher education: a literature review of online teaching and learning practices. Eur. J. Teach. Educ. 43, 466–487. doi: 10.1080/02619768.2020.1821184

[ref14] Chang C.-F. Gröschner A. Hall N. C. Alles M. Seidel T. (2018). Exploring teachers’ emotions via nonverbal behavior during video-based teacher professional development. AERA Open 4:233285841881985. doi: 10.1177/2332858418819851

[ref15] Chango W. Cerezo R. Sanchez-Santillan M. Azevedo R. Romero C. (2021). Improving prediction of students’ performance in intelligent tutoring systems using attribute selection and ensembles of different multimodal data sources. J. Comput. High. Educ. 33, 614–634. doi: 10.1007/s12528-021-09298-8

[ref16] Chen Y. Chen N.-S. Tsai C.-C. (2009). The use of online synchronous discussion for web-based professional development for teachers. Comput. Educ. 53, 1155–1166. doi: 10.1016/j.compedu.2009.05.026

[ref17] Cleary T. J. Zimmerman B. J. (2012). “A cyclical self-regulatory account of student engagement-theoretical foundations and applications” in Handbook of Research on Student Engagement. eds. Christenson S. L. Wylie A. Reschly C. (Boston, MA: Springer Science+Business Media), 237–257.

[ref18] Cooper N. Burgess A. Croft R. Gruzelier J. (2006). Investigating evoked and induced electroencephalogram activity in task-related alpha power increases during an internally directed attention task. Neuroreport 17, 205–208. doi: 10.1097/01.wnr.0000198433.29389.54, PMID: 16407772

[ref19] Costa E. B. Fonseca B. Santana M. A. de Araújo F. F. Rego J. (2017). Evaluating the effectiveness of educational data mining techniques for early prediction of students' academic failure in introductory programming courses. Comput. Hum. Behav. 73, 247–256. doi: 10.1016/j.chb.2017.01.047

[ref20] Cranford K. N. Tiettmeyer J. M. Chuprinko B. C. Jordan S. Grove N. P. (2014). Measuring load on working memory: the use of heart rate as a means of measuring chemistry students’ cognitive load. J. Chem. Educ. 91, 641–647. doi: 10.1021/ed400576n

[ref21] Creemers B. Kyriakides L. Panayiotis A. (2012). Teacher Professional Development for Improving Quality of Teaching. New York, USA: Springer Publishing.

[ref22] Cui Y. Song X. Hu Q. Li Y. Sharma P. Khapre S. (2022). Human-robot interaction in higher education for predicting student engagement. Comput. Electr. Eng. 99:107827. doi: 10.1016/j.compeleceng.2022.107827

[ref23] Dabbagh N. Kitsantas A. (2004). Supporting self-regulation in student-centered web-based learning environments. EdMedia + Innovate Learn. Online 2022 3, 40–47. Available at: https://www.learntechlib.org/primary/p/4104/

[ref24] Deng R. Benckendorff P. Gannaway D. (2020). Learner engagement in MOOCs: Scale development and validation. Br. J. Educ. Technol. 51, 245–262. doi: 10.1111/bjet.12810

[ref25] D’Mello S. Dieterle E. Duckworth A. (2017). Advanced, analytic, automated (AAA) measurement of engagement during learning. Educ. Psychol. 52, 104–123. doi: 10.1080/00461520.2017.1281747, PMID: 29038607 PMC5640167

[ref26] Dubovi I. (2022). Cognitive and emotional engagement while learning with VR: the perspective of multimodal methodology. Comput. Educ. 183:104495. doi: 10.1016/j.compedu.2022.104495

[ref27] Emerson A. Cloude E. B. Azevedo R. Lester J. (2020). Multimodal learning analytics for game-based learning. Br. J. Educ. Technol. 51, 1505–1526. doi: 10.1111/bjet.12992

[ref28] Fredricks J. A. Blumenfeld P. C. Paris A. H. (2004). School engagement: potential of the concept, state of the evidence. Rev. Educ. Res. 74, 59–109. doi: 10.3102/00346543074001059

[ref30] Giannakos M. N. Sharma K. Pappas I. O. Kostakos V. Velloso E. (2019). Multimodal data as a means to understand the learning experience. Int. J. Inf. Manag. 48, 108–119. doi: 10.1016/j.ijinfomgt.2019.02.003

[ref001] Greene B. A. (2015). Measuring cognitive engagement with self-report scales: reflections from over 20 years of research. Educ. Psychol. 50, 14–30. doi: 10.1080/00461520.2014.989230

[ref31] Hassib M. Khamis M. Friedl S. Schneegaß S. Alt F. (2017). “Brainatwork: logging cognitive engagement and tasks in the workplace using electroencephalography,” in Proceedings of the 16th International Conference on Mobile and Ubiquitous Multimedia (MUM’17). New York, NY, USA: Association for Computing Machinery, 305–310.

[ref32] Helme S. Clarke D. (2001). Identifying cognitive engagement in the mathematics classroom. Math. Educ. Res. J. 13, 133–153. doi: 10.1007/BF03217103

[ref33] Holmes S. R. Reinke W. M. Herman K. C. David K. (2021). An examination of teacher engagement in intervention training and sustained intervention implementation. Sch. Ment. Heal. 14, 63–72. doi: 10.1007/s12310-021-09457-3

[ref34] Kang D. Oh S. (2020). Balanced training/test set sampling for proper evaluation of classification models. Intell. Data Anal. 24, 5–18. doi: 10.3233/IDA-194477

[ref35] Ke F. Xie K. (2009). Toward deep learning for adult students in online courses. Internet High. Educ. 12, 136–145. doi: 10.1016/j.iheduc.2009.08.001

[ref36] Kosmyna N. Maes P. (2019). AttentivU: an EEG-based closed-loop biofeedback system for real-time monitoring and improvement of engagement for personalized learning. Sensors (Basel) 19:5200. doi: 10.3390/s19235200, PMID: 31783646 PMC6929136

[ref37] Larmuseau C. Cornelis J. Lancieri L. Desmet P. Depaepe F. (2020). Multimodal learning analytics to investigate cognitive load during online problem solving. Br. J. Educ. Technol. 51, 1548–1562. doi: 10.1111/bjet.12958

[ref38] Li S. Lajoie S. P. (2021). Cognitive engagement in self-regulated learning: an integrative model. Eur. J. Psychol. Educ. 37, 833–852. doi: 10.1007/s10212-021-00565-x

[ref39] Liao W. Xu W. Kong S. Ahmad F. Liu W. (2019). “A two-stage method for hand-raising gesture recognition in classroom,” in *Proceedings of the 2019 8th International Conference on Educational and Information Technology*. 38–44.

[ref40] Lin P.-C. Hou H.-T. Wu S.-Y. Chang K.-E. (2014). Exploring college students' cognitive processing patterns during a collaborative problem-solving teaching activity integrating Facebook discussion and simulation tools. Internet High. Educ. 22, 51–56. doi: 10.1016/j.iheduc.2014.05.001

[ref41] Liu I. F. Chen M. C. Sun Y. S. Wible D. Kuo C.-H. (2010). Extending the TAM model to explore the factors that affect intention to use an online learning community. Comput. Educ. 54, 600–610. doi: 10.1016/j.compedu.2009.09.009

[ref42] Liu D. Zhang H. (2021). Developing a new model for understanding teacher satisfaction with online learning. SAGE Open 11:215824402110364. doi: 10.1177/21582440211036440

[ref43] Mayordomo R. M. Espasa A. Guasch T. Martínez-Melo M. (2022). Perception of online feedback and its impact on cognitive and emotional engagement with feedback. Educ. Inf. Technol. 27, 7947–7971. doi: 10.1007/s10639-022-10948-2

[ref44] Moreno-Marcos P. M. Muñoz-Merino P. J. Maldonado-Mahauad J. Pérez-Sanagustín M. Alario-Hoyos C. Delgado Kloos C. (2020). Temporal analysis for dropout prediction using self-regulated learning strategies in self-paced MOOCs. Comput. Educ. 145:103728. doi: 10.1016/j.compedu.2019.103728

[ref45] Nami F. (2021). Developing in-service teachers’ pedagogical knowledge of CALL through project-oriented tasks: the case of an online professional development course. ReCALL 34, 110–125. doi: 10.1017/s0958344021000148

[ref46] Nasir M. K. M. Ngah A. H. (2022). The sustainability of a Community of Inquiry in online course satisfaction in virtual learning environments in higher education. Sustainability 14:9633. doi: 10.3390/su14159633

[ref47] Niedermeyer E. da Silva F. L. (2005). Electroencephalography: Basic Principles, Clinical Applications, and Related Fields, vol. 99. Philadelphia, USA: Lippincott Williams & Wilkins, 107827.

[ref48] Ning H. K. Downing K. (2011). The interrelationship between student learning experience and study behaviour. Higher Educ. Res. Dev. 30, 765–778. doi: 10.1080/07294360.2010.539598

[ref49] Noldus (2019). Available at: https://www.noldus.com/applications/emotion-analysis (Accessed December 1, 2022).

[ref50] Olsen J. K. Sharma K. Rummel N. Aleven V. (2020). Temporal analysis of multimodal data to predict collaborative learning outcomes. Br. J. Educ. Technol. 51, 1527–1547. doi: 10.1111/bjet.12982

[ref51] Parsons S. Hutchison A. Hall L. Parsons A. Ives S. Leggett A. (2019). U.S. teachers’ perceptions of online professional development. Teach. Teach. Educ. 82, 33–42. doi: 10.1016/j.tate.2019.03.006

[ref52] Philipsen B. Tondeur J. Pareja Roblin N. Vanslambrouck S. Zhu C. (2019). Improving teacher professional development for online and blended learning: a systematic meta-aggregative review. Educ. Technol. Res. Dev. 67, 1145–1174. doi: 10.1007/s11423-019-09645-8

[ref53] Powell C. G. Bodur Y. (2019). Teachers’ perceptions of an online professional development experience: implications for a design and implementation framework. Teach. Teach. Educ. 77, 19–30. doi: 10.1016/j.tate.2018.09.004

[ref54] Psaltis A. Apostolakis K. C. Dimitropoulos K. Daras P. (2018). Multimodal student engagement recognition in prosocial games. IEEE Trans. Games 10, 292–303. doi: 10.1109/TCIAIG.2017.2743341

[ref55] Richardson J. C. Alsup J. (2015). From the classroom to the keyboard: how seven teachers created their online teacher identities. Int. Rev. Res. Open Distrib. Learn. 16, 142–167. doi: 10.19173/irrodl.v16i1.1814

[ref57] Rogers P. (2001). Traditions to transformations: the forced evolution of higher education. AACE J. 9, 47–60.

[ref58] Ross J. D. (2011). Online Professional Development: Design, Deliver, Succeed! Thousand Oaks, California: Corwin Press.

[ref59] Ross S. M. Morrison G. R. (2004). “Experimental research methods,” in *Handbook of Research on Educational Communications and Technology*. Mahwah: Lawrence Erlbaum Associates, *Vol. 2*. 1021–1043.

[ref60] Sharma K. Giannakos M. (2020). Multimodal data capabilities for learning: what can multimodal data tell us about learning? Br. J. Educ. Technol. 51, 1450–1484. doi: 10.1111/bjet.12993

[ref61] Sharma K. Papamitsiou Z. Olsen J. K. Giannakos M. (2020). “Predicting learners' effortful behaviour in adaptive assessment using multimodal data,” in *Proceedings of the Tenth International Conference on Learning Analytics & Knowledge*.

[ref62] Sinatra G. M. Heddy B. C. Lombardi D. (2015). The challenges of defining and measuring student engagement in science. Educ. Psychol. 50, 1–13. doi: 10.1080/00461520.2014.1002924

[ref63] Soleymani M. Asghari-Esfeden S. Fu Y. Pantic M. (2016). Analysis of EEG signals and facial expressions for continuous emotion detection. IEEE Trans. Affect. Comput. 7, 17–28. doi: 10.1109/TAFFC.2015.2436926

[ref65] Taub M. Sawyer R. Lester J. Azevedo R. (2019). The impact of contextualized emotions on self-regulated learning and scientific reasoning during learning with a game-based learning environment. Int. J. Artif. Intell. Educ. 30, 97–120. doi: 10.1007/s40593-019-00191-1

[ref66] Teräs H. (2014). Collaborative online professional development for teachers in higher education. Prof. Dev. Educ. 42, 258–275. doi: 10.1080/19415257.2014.961094

[ref67] Terzis V. Moridis C. Economides A. (2013). Measuring instant emotions based on facial expressions during computer-based assessment. Pers. Ubiquit. Comput. 17, 43–52. doi: 10.1007/s00779-011-0477-y

[ref68] Törmänen T. Järvenoja H. Mänty K. (2021). Exploring groups’ affective states during collaborative learning: what triggers activating affect on a group level? Educ. Technol. Res. Dev. 69, 2523–2545. doi: 10.1007/s11423-021-10037-0

[ref69] Ulmanen S. Soini T. Pietarinen J. Pyhältö K. (2016). Students’ experiences of the development of emotional engagement. Int. J. Educ. Res. 79, 86–96. doi: 10.1016/j.ijer.2016.06.003

[ref70] Vanneste P. Oramas J. Verelst T. Tuytelaars T. Raes A. Depaepe F. (2021). Computer vision and human behaviour, emotion and cognition detection: a use case on student engagement. Mathematics 9:287. doi: 10.3390/math9030287

[ref71] Wang Y. Cao Y. Gong S. Wang Z. Li N. Ai L. (2022). Interaction and learning engagement in online learning: the mediating roles of online learning self-efficacy and academic emotions. Learn. Individ. Differ. 94:102128. doi: 10.1016/j.lindif.2022.102128

[ref72] Wang Y. Chen N.-S. Levy M. (2010). Teacher training in a synchronous cyber face-to-face classroom: characterizing and supporting the online teachers' learning process. Comput. Assist. Lang. Learn. 23, 277–293. doi: 10.1080/09588221.2010.493523

[ref73] Whitehill J. Serpell Z. Lin Y. C. Foster A. Movellan J. R. (2014). The faces of engagement: automatic recognition of student engagement from facial expressions. IEEE Trans. Affect. Comput. 5, 86–98. doi: 10.1109/TAFFC.2014.2316163

[ref74] Wigfield A. Eccles J. Fredricks J. Simpkins S. Roeser R. Schiefele U. (2015). “Development of achievement motivation and engagement,” in Handbook of child psychology and developmental cience:socioemotional processes. eds. Lamb M. E. Lerner R. M. (John Wiley & Sons, Inc.) 1–44.

[ref75] Wolff C. E. Jarodzka H. van den Bogert N. Boshuizen H. P. A. (2016). Teacher vision: expert and novice teachers’ perception of problematic classroom management scenes. Instr. Sci. 44, 243–265. doi: 10.1007/s11251-016-9367-z

[ref76] Ye J.-M. Zhou J. (2022). Exploring the relationship between learning sentiments and cognitive processing in online collaborative learning: a network analytic approach. Internet High. Educ. 55:100875. doi: 10.1016/j.iheduc.2022.100875

[ref77] Zheng K. Yang D. Liu J. Cui J. (2020). Recognition of teachers’ facial expression intensity based on convolutional neural network and attention mechanism. IEEE Access 8, 226437–226444. doi: 10.1109/ACCESS.2020.3046225

